# The impact of fixed orthodontic appliances on oral microbiome dynamics in Japanese patients

**DOI:** 10.1038/s41598-020-78971-2

**Published:** 2020-12-15

**Authors:** Isamu Kado, Junzo Hisatsune, Keiko Tsuruda, Kotaro Tanimoto, Motoyuki Sugai

**Affiliations:** 1 Department of Orthodontics and Craniofacial Developmental Biology, Hiroshima University Graduate School of Biomedical and Health Sciences, Hiroshima, Japan; 2 Department of Antimicrobial Resistance, Hiroshima University Graduate School of Biomedical and Health Sciences, Hiroshima, Japan; 3 Department of Oral Epidemiology, Hiroshima University Graduate School of Biomedical and Health Sciences, Hiroshima, Japan; 4 Antimicrobial Resistance Research Center, National Institute of Infectious Diseases, 4-2-1 Aoba-cho, Higashimurayama city, Tokyo 189-0002 Japan

**Keywords:** Microbiology, Bacteriology, Microbial communities

## Abstract

Fixed orthodontic appliances are common and effective tools to treat malocclusion. Adverse effects of these appliances, such as dental caries and periodontitis, may be associated with alteration of the microbiome. This study investigated the impact of these appliances on the dynamics of the oral microbiome. Seventy-one patients were selected. Supragingival plaque samples were collected before placement (T0) and six months after placement (T1). Saliva samples were collected at T0 and T1, and then when appliance removal (T2). Microbial DNA was analyzed by 16S rRNA meta-sequencing. The diversity analysis indicated dynamic changes in the structure of the oral microbiome. Taxonomic analysis at phylum level showed a significant increase in Bacteroidetes and Saccharibacteria (formally TM7) and decrease in Proteobacteria and Actinobacteria over time, in both plaque and saliva. Genus level analysis of relative abundance indicated a significant increase in anaerobic and facultative anaerobes in both plaque and saliva. Fixed orthodontic appliances induced measurable changes in the oral microbiome. This was characterized by an increase in relative abundance of obligate anaerobes, including periodontal pathogens. It can be concluded that this dysbiosis induced by fixed orthodontic appliances is likely to represent a transitional stage in the shift in microbiome from healthy to periodontitis.

## Introduction

The mouth harbors more than 700 bacterial species, constituting one of the most diverse bacterial communities in the human body^1^. The mouth comprises complex structures of hard and soft tissue, such as teeth, tongue, gingiva, and palate; unique variation in oral microbiome structure is observed according to the different surface properties^2,3^.

Fixed orthodontic appliances are a common and effective tool used to treat malocclusion, but can be associated with secondary effects, such as a change of microbiome and subsequent infections. The complicated undercut shape of orthodontic appliances makes teeth cleaning more difficult and induces plaque accumulation as well as need for restorations^4–6^. Therefore, it has been suggested that the risk of white-spot lesions, dental caries, and periodontal complication are due to the change in oral microbiome. The incidence and prevalence rates of white-spot lesions in patients undergoing orthodontic treatment are high, but these are incipient carious lesions that can be remineralized by application of fluoride^7^. Previous studies have suggested that an increase in *Streptococcus mutans* bacterial counts, generally regarded as a major risk factor for dental caries, is associated with placement of fixed orthodontic appliances^8–11^.

It is understood that anaerobic microorganisms in plaque play a key role in the initiation and acceleration of periodontal diseases. Periodontal pathogens, such as *Fusobacterium, Treponema, and Porphyromonas* spp., have been detected in dental plaque around orthodontic appliances^12^. Furthermore, the frequency of *Tannerella forsythia, Campylobacter rectus,* and *Prevotella nigrescens* increased after placement of orthodontic appliances^13^. Severe clinical attachment loss during orthodontic treatment has also been reported^14^. These reports suggest that fixed orthodontic appliances may change the oral microbiome and have the potential to shift the bacterial ecosystem toward a pathogenic state. However, most previous reports have focused on only specific species and very few have focused on the microbiome and its dynamics, including unculturable bacteria, following orthodontic treatment.

Conventional methods of bacteriological identification, such as cultivation, present limitations to the analysis of microbial community structure and diversity, because the human bacterial flora contains many unculturable species. Emergence of Next Generation Sequencing (NGS) technology has enabled analysis and comparison of bacterial composition, including unculturable bacteria, with unprecedented depth compared to previous methodologies^15^. However, there are few reports about the relationship between orthodontic treatment and the dynamics of oral microbiome. In previous report using NGS, periodontal pathogens were highest during orthodontic treatment, but the data was confined about plaque^16^.

The aim of this study was to assess the changes in oral microbiome dynamics caused by fixed orthodontic appliances using 16S rRNA gene meta-sequencing of supragingival plaque and saliva.

## Results

### Operational taxonomic unit clustering and trim report

In total, 13,506,556 reads were generated from 144 samples, with an average length of 300.4. After sequence trimming, 8,284,029 high quality reads remained, with an average length of 222.5 bp. In plaque samples, 44 samples were successfully analyzed both at T0 and T1. In saliva samples, 16, 23, and 17 samples were successfully analyzed at T0, T1 and T2 respectively. We detected 983 OTUs in total, with an average of 341 OTUs per sample. The number of OTUs increased according to time course both in plaque (T0, 312 and T1, 321) and saliva samples (T0, 329; T1, 366; and T2, 376) though the difference was not significant.

### Alpha and beta diversity

Alpha diversity, a measure of microbial community evenness and richness in each sample, was calculated and compared between time points (2 for plaque samples and 3 for saliva samples; Supplementary Fig. 1). The difference in number of OTUs in each sample relates to bacterial community diversity and the difference in number of reads at the end of clustering relates to diversity of sample DNA concentrations. Beta diversity, a measure of the variation of microbial communities between samples, was calculated and compared using Principal Coordinates Analysis (PCoA), based on the Jaccard index.

In plaque samples, bacterial diversity at T1 (321 ± 60 OTUs; range 214–430) was slightly more diverse than at T0 (312 ± 69; range 189–465; Supplementary Fig. 1a). There was no significant difference between upper and lower teeth at either T0 or T1. In saliva samples, there were no visible changes of bacterial community diversity between T0 (329 ± 54; range 240–409), T1 (366 ± 62; 271–481), and T2 (376 ± 60; range 214–430; appendix Supplementary Fig. 1b).

**Figure 1:**
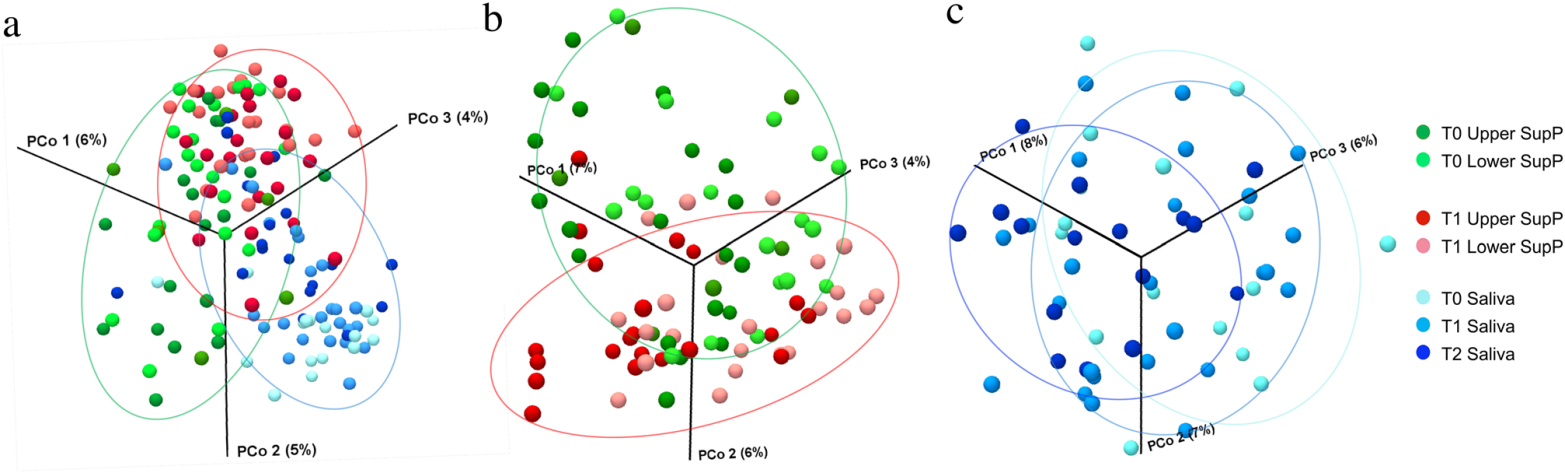
Beta diversity of the oral microbiome following placement of fixed orthodontic devices. Beta diversity Jaccard index, based on principal coordinates analysis (PCoA) of all samples (**a**), supragingival samples (**b**), and saliva samples (**c**). Green dots and red dots represent T0 and T1 samples, respectively and dark and light colors represent upper and lower teeth, respectively. In saliva samples, dark, middle, and light blue dots represent T0, T1, and T2 samples, respectively. *T0* time point prior to placement of fixed orthodontic appliance, *T1* time point approximately 6 months after the start of orthodontic treatment, *T2* time point after appliance removal (average, 40 months after placement). These images were generated by CLC Genomics Workbench ver.9 (QIAGEN, Venlo, Netherlands) (https://www.qiagen.com/jp/products/discovery-and-translational-research/next-generation-sequencing/informatics-and-data/analysis-and-visualization/clc-genomics-workbench/#orderinginformation).

Combined analysis of the beta diversity of plaque and saliva showed that they consisted of different bacterial communities (Fig. 1a). Comparison of T0 with T1 plaque samples indicates that these two types of sample were apart from each other (Fig. 1b). We did not observe any obvious shifts in diversity between saliva samples at T0, T1, or T2 (Fig. 1c).

### Taxonomic analysis

**Figure 2:**
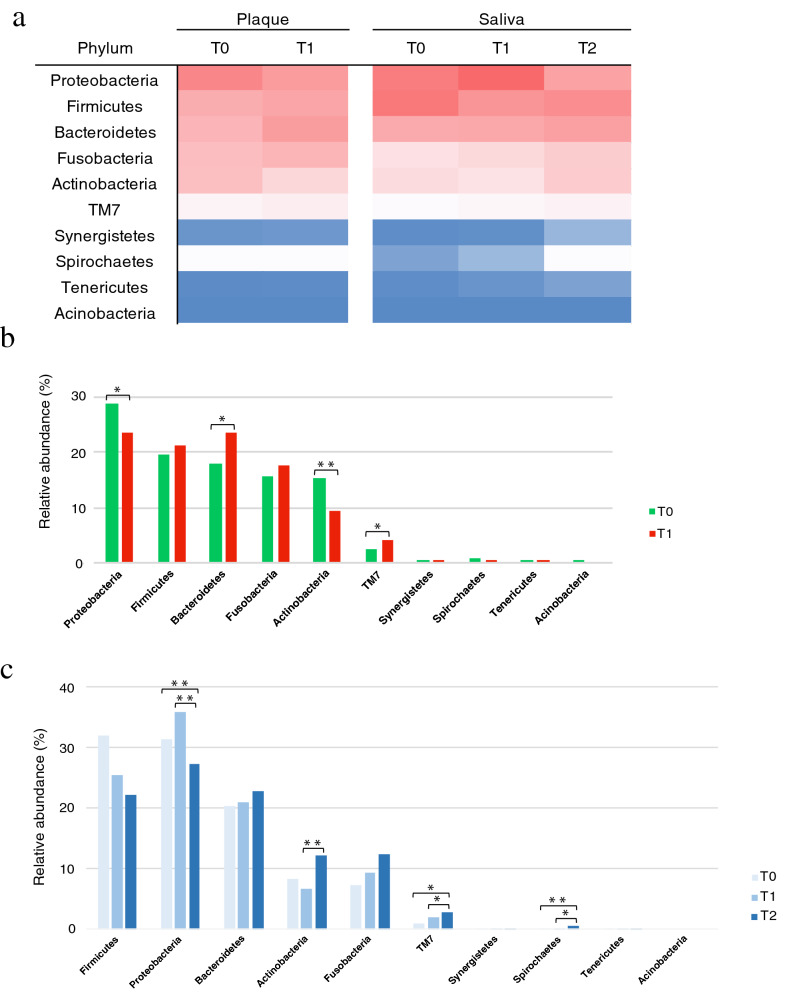
Bacterial population structure at phylum level. (**a**) Heat map of the relative abundance of bacteria from supragingival plaque and saliva, at phylum level, at each time point. (**b**) Relative abundance of bacteria in supragingival plaque, at phylum level. The green and red bars represent T0 and T1, respectively. (**c**) Relative abundance of bacteria in saliva, at phylum level. The dark, middle and light blue bars express T0, T1, and T2 respectively. Significant differences are indicated as *p < 0.05 and **p < 0.01 by Mann Whitney U (**b**) and Kruskal–Wallis (**c**) tests. *T0* time point prior to placement of fixed orthodontic appliance, *T1* time point approximately 6 months after the start of orthodontic treatment, *T2* time point after appliance removal (average, 40 months after placement).

Bacterial structure, at phylum level, is shown in Fig. 2a as a heat map. The predominant phyla were Proteobacteria, Firmicutes, Bacteroidetes, Fusobacteria, and Actinobacteria, both in plaque and saliva. Phylum level bacterial distribution based on relative abundance in plaque samples is shown in Fig. 2b. The most dominant phylum both at T0 and T1 was Proteobacteria (28.74% and 23.56%, respectively), although Bacteroidetes distribution at T1 (23.44%) was similar to that of Proteobacteria. Proteobacteria and Actinobacteria significantly decreased at T1 (28.74–23.56% and 15.11–9.39%, respectively), whereas Bacteroidetes and TM7 significantly increased at T1 (17.78–23.44% and 2.59–4.08%, respectively).

Phylum level bacterial distribution based on relative abundance in saliva samples is shown in Fig. 2c. Actinobacteria significantly increased from T1 (6.68%) to T2 (12.07%). TM7 significantly increased, in a time-dependent manner, from T0 (0.87%) to T1 (1.99%) and T2 (2.77%) and Spirochaetes demonstrated a similar trend (0.08%, 0.15%, and 0.64%, respectively). Fusobacteria and Bacteroidetes distribution also increased with time but the difference was not significant. Proteobacteria remained similar at T0 (31.22%) and T1 (35.75%) and significantly decreased at T2 (27.13%).

**Figure 3:**
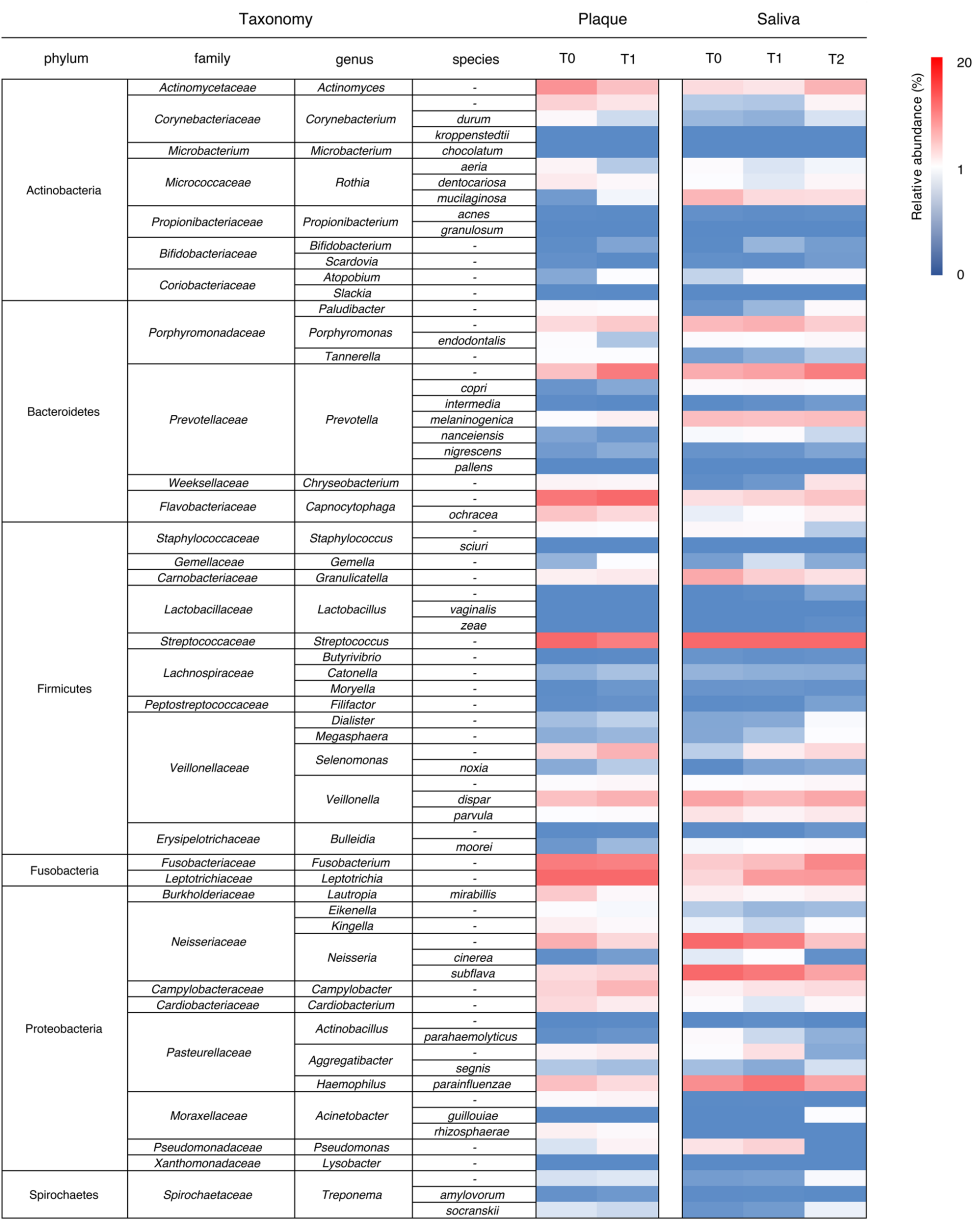
Bacterial population structure at genus or species level. Heat map of the relative abundance of bacteria in supragingival plaque and saliva, at genus or species level, at each time point. *T0* time point prior to placement of fixed orthodontic appliance, *T1* time point approximately 6 months after the start of orthodontic treatment, *T2* time point after appliance removal (average, 40 months after placement).

The overall microbiome in plaque and saliva is shown in Fig. 3 at phylum, family, genus, and species level. Predominant microbial distribution (> 5.0% of the relative abundance, dark blue scale in the heat map) in plaque samples at genus level were *Leptotrichia, Streptococcus, Capnocytophaga, Fusobacterium, Prevotella*, and *Actinomyces.* In saliva samples, predominant genera were *Streptococcus, Neisseria, Haemophilus, Prevotella*, and *Veillonella. Capnocytophaga, Fusobacterium*, and *Leptotrichia* spp. were more relatively abundant in supragingival plaque than in saliva. Conversely, *Neisseria* and *Haemophilus* spp. were more abundant in saliva.

Increased bacterial abundance in supragingival plaque with time are shown in Table 1a. Relative abundance of *Prevotella*, *Porphyromonas, Capnocytophaga, Parvimonas*, *and Selenomonas* spp., which are implicated in periodontal diseases, were significantly higher at T1 than T0. There was no significant difference in other bacteria, unrelated to periodontitis, between T0 and T1. Most of the bacteria which demonstrated increased abundance over time were obligate anaerobes.

**Table 1:** List of the bacteria that showed dynamics.

(a)							
Genus	Species	RA at T0 (%)	RA at T1 (%)	*p*-value	Oxygen demand	Relevant pathological conditions	
*Atopobium*	*–*	0.12	0.44	0.002**	Facultative anaerobic	Periodontitis, vaginosis	
*Solobacterium*	*Moorei*	0.05	0.18	0.006**	Obligate anaerobic	Periodontitis, dentoalveolar abscess	
*Campylobacter*	*–*	2.77	4.48	0.006**	Facultative anaerobic	Periodontitis	
*Capnocytophaga*	*–*	3.63	8.94	0.001**	Facultative anaerobic	Periodontitis, animal bite wounds	
*Gemella*	*–*	0.16	0.45	0.047*	Facultative anaerobic	Endocarditis	
*Leptotrichia*	*–*	10.83	14.04	0.112	Obligate anaerobic	Endocarditis	
*Moryella*	*–*	0.01	0.05	0.002**	Obligate anaerobic	–	
*Parvimonas*	*Micra*	0.01	0.09	0.001**	Facultative anaerobic	Periodontitis, septic arthritis	
*Porphyromonas*	*–*	2.42	3.35	0.070	Obligate anaerobic	Periodontitis	
*Prevotella*	*–*	3.81	7.69	0.001**	Obligate anaerobic	Periodontitis	
*Prevotella*	*Melaninogentica*	0.52	1.18	0.048*	Obligate anaerobic	–	
*Selenomonas*	*–*	2.54	4.61	0.001**	Obligate anaerobic	–	
*Selenomonas*	*Noxia*	0.12	0.25	0.036*	Obligate anaerobic	Periodontitis	
*Veillonella*	*Disper*	3.85	4.69	0.225	Obligate anaerobic	–	
*Veillonella*	*Parvula*	0.44	0.56	0.304	Obligate anaerobic	Periodontitis, meningitis	

(b)							
Genus	Species	RA at T0	RA at T1	*p*-value	oxygen demand	Relevant pathological conditions	
*Acinetobacter*	*Rhizosphaerae*	1.16	0.69	0.108	Aerobic	Nosocomial infections	
*Actinobacillus*	–	0.70	0.01	0.005**	Facultative anaerobic	Endocarditis, periodontitis	
*Actinomyces*	–	6.40	3.82	0.007**	Facultative anaerobic	Dentoalveolar abscess	
*Corynebacterium*	–	2.86	1.80	0.041*	Aerobic	–	
*Haemophilus*	*Parainfluenzae*	3.88	2.35	0.046*	Facultative anaerobic	Pneumonia	
*Kingella*	–	1.23	0.67	0.039*	Aerobic	Endocarditis, septic arthritis	
*Lautropia*	*Mirabillis*	3.33	0.72	0.001**	Facultative anaerobic	Cystic fibrosis	
*Neisseria*	–	4.80	2.51	0.009**	Aerobic	–	
*Rothia*	*Aeria*	0.95	0.24	0.407	Facultative anaerobic	Endocarditis	
*Rothia*	*Dentocariosa*	1.49	0.77	0.049*	Facultative anaerobic	Dental caries, endocarditis	
*Streptococcus*	–	9.47	7.53	0.091	Facultative anaerobic	–	

(c)							
Genus	Species	RA at T0	RA at T1	RA at T2	*p*-value	oxygen demand	Relevant pathological conditions
*Atopobium*	–	0.28	0.64	0.65	0.046*	Facultative anaerobic	Periodontitis, vaginosis
*Campylobacter*	–	1.08	1.85	2.32	0.008**	Facultative anaerobic	Periodontitis
*Capnocytophaga*	–	2.16	2.73	3.63	0.245	Facultative anaerobic	Periodontitis, animal bite wounds
*Capnocytophaga*	*Ochracea*	0.38	0.53	1.17	0.002**	Facultative anaerobic	Periodontitis, dental caries
*Fusobacterium*	–	3.25	4.02	7.04	0.001**	Obligate anaerobic	Periodontitis, colon cancer
*Leptotrichia*	–	2.58	5.94	6.03	0.001**	Obligate anaerobic	Endocarditis
*Paludibacter*	–	0.04	0.17	0.67	0.001**	Obligate anaerobic	–
*Prevotella*	–	4.94	5.56	7.57	0.032*	Obligate anaerobic	Periodontitis
*Prevotella*	*Nigrescens*	0.04	0.04	0.10	0.035*	Obligate anaerobic	Periodontitis, extraoral infections
*Selenomonas*	–	0.26	1.34	2.47	0.001**	Obligate anaerobic	Periodontitis
*Selenomonas*	*Noxia*	0.01	0.10	0.12	0.004**	Obligate anaerobic	Periodontitis
*Tannerella*	*-*	0.08	0.14	0.24	0.023*	Obligate anaerobic	Periodontitis

(d)							
Genus	Species	RA at T0	RA at T1	RA at T2	*p*-value	Oxygen demand	Relevant pathological conditions
*Granulicatella*	–	5.14	3.04	2.05	0.046*	Facultative anaerobic	Endocarditis
*Neisseria*	–	9.16	7.58	3.62	0.054	Aerobic	–
*Neisseria*	*Subflava*	9.94	7.86	5.48	0.162	aerobic	Meningitis
*Rothia*	*Mucilaginosa*	4.40	2.37	2.25	0.832	Facultative anaerobic	Endocarditis
*Streptococcus*	–	15.97	12.86	11.73	0.220	Facultative anaerobic	–

(a) Increased bacteria in plaque with time and (b) decreased bacteria in plaque with time at genus or species level. Significant differences are indicated as bold letters and *p < 0.05, **p < 0.01 by Mann–Whitney U test. (c) Increased bacteria in saliva and (d) decreased bacteria in saliva with time at genus or species level. Significant differences are indicated as bold letters and *p < 0.05, **p < 0.01 by Kruskal–Wallis test.

Bacteria which decreased over time in supragingival plaque are listed in Table 1b. In accordance with the phylum level results, many genera belonging to Actinobacteria, such as *Actinomyces, Corynebacterium*, and *Rothia*, and Proteobacteria, such as *Neisseria*, *Haemophilus*, and *Lautropia*, were significantly lower at T1 than at T0. All bacteria that decreased with time were aerobes or facultative anaerobes. *Streptococcus* spp., including cariogenic group decreased from T0 (9.47%) to T1 (7.53%), although the difference was not significant.

Increased bacterial abundance in saliva over time is shown in Table 1c and reflect the results seen in plaque. Periodontal pathogens such as *Prevotella, Porphyromonas, Capnocytophaga, Tannerella, Fusobacterium, Selenomonas*, and *Atopobium* spp. significantly increased stepwise from T0 to T2. All the other bacteria that increased in saliva were facultative or obligate anaerobes.

Bacteria in saliva that decreased over time are shown in Table 1d. *Neisseria*, one of the core genera in saliva, decreased stepwise from T0 (9.16%) to T1 (7.58%) and T2 (3.62%) and *Streptococcus* decreased from T0 (15.97%) to T1 (12.86%) and T2 (11.73%), although these differences were not significant. All bacteria that decreased with time were aerobes or facultative anaerobes, consistent with the results in plaque.

## Discussion

Numerous studies of the relationship between oral bacteria and orthodontic treatment have been performed via conventional methods, such as cultivation, and these studies have focused on specific types of bacteria. For example, increases in the incidence of dental caries and *S. mutans* bacterial counts were seen with fixed orthodontic appliances^17,18^. Similarly, placement of orthodontic appliances influenced clinical parameters and colonization of periodontal pathogenic bacteria such as *P. gingivalis*, *P. intermedia, P. nigrescens*, *and F. nucleatum*^19–21^. In this study, the number of detected OTUs slightly increased with time both in plaque and saliva, although there was no significant difference, suggesting that bacterial structure may have become more diverse after application of orthodontic appliances. The relative segregation in PCoA demonstrated discrepancies between bacterial structures at different time points, especially in plaque samples (Fig. 1a–c). There was no obvious difference between samples from upper and lower teeth.

Analysis of relative abundance at phylum level revealed more detail on bacterial distribution (Fig. 2a–c) such as the significant decrease in Proteobacteria and Actinobacteria in plaque. A wide variety of the normal flora found in the gut and oral cavity belong to the phylum Proteobacteria, while Actinobacteria represent important members of the environmental microbiome. The significant decrease of Actinobacteria after the placement of fixed orthodontic appliances have been reported previously^16^. Conversely, Bacteroidetes and Saccharibacteria (formally TM7), both anaerobes, significantly increased over time. Bacteroidetes are widely distributed in gut, oral cavity, and skin and include periodontal pathogenic bacteria, such as *P. gingivalis* and *P. intermedia*. Laboratory culture of Saccharibacteria has so far been impossible, here NGS analysis allowed us to detect this phylum. Saccharibacteria lives on the surface of its host bacterium. They affect oral microbial ecology by modulating the microbiome structure hierarchy and functionality through affecting the host’s physiology and the relative abundance of the host via direct killing^22^. Both in plaque and saliva samples, Saccharibacteria abundance significantly increased over time. The average age of the population sampled at T2 was different from that at T0 and T1, therefore we believe that this is likely also linked to the differences in microbiome, including Saccharibacteria abundance, observed at T2. Some reports suggest that Saccharibacteria increase with advancing age and have a role in initiating periodontitis^23,24^.

The genera *Prevotella, Capnocytophaga, Atopobium*, *Selenomonas*, and *Campylobacter* significantly increased with time in both plaque and saliva (Table 1a,c). The genus *Prevotella*, of the phylum Bacteroidetes, is comprised of obligate anaerobes and well-known pathogens of periodontal diseases^25^. Tanner et al. reported the genus *Prevotella* showed higher detection rate in plaque at high gingivitis group of orthodontic patients^26^. *P. intermedia,* one of the red-complex bacteria^27^, was observed to increase after the placement of an orthodontic appliance^19^ and then decrease after appliance removal in one study, but Choi et al. did not observe significant decrease following removal^28^. Guo et al. reported that *P. intermedia* showed significant increase not in molar area but in incisor area^29^. At species level, *Prevotella melaninogentica* and *Prevotella nigrescens*, which are also implicated in periodontitis, significantly increased in plaque and saliva, respectively (Table 1a,c). The genera *Capnocytophaga, Campylobacter, Atopobium*, and *Selenomonas* are facultative anaerobes. Interestingly, these genera are implicated in pathogenesis of periodontal diseases^30–35^. The genus *Selenomonas* were reported to increase after six and twelve weeks from the placement of orthodontic appliances in previous study^16^. The genus *Porphyromonas* (including *P. gingivalis*) which is obligate anaerobe and regarded as a major pathogen of periodontitis with highly proteolytic activity became higher in abundance during orthodontic treatment in plaque samples although there was no significant difference^36^. *Aggregatibacter actinomycetemcomitans,* one of the pathogens of periodontitis, was reported to significantly increase three or six months after the placement of orthodontic appliances and remained higher level after removal^37,38^. These data suggest that the bacteria that significantly increased in relative abundance, in both plaque and saliva, were obligate and facultative anaerobes; notably most have been reported to be associated with periodontal diseases. No significant increase in the relative abundance of aerobes were detected after placement of appliances. These results strongly suggest that fixed orthodontic appliances alter the oral microbiome towards an anaerobic and periodontopathogenic state.

Abundance of organisms belonging to the genera *Actinobacillus, Actinomyces, Corynebacterium, Kingella*, and *Neisseria* and the species *Haemophilus parainfluenzae, Lautropia mirabillis*, and *Rothia dentocariosa* significantly decreased over time in plaque samples. Regarding *Actinomyces*, some studies reported that they significantly increase in plaque by orthodontic treatment. Koopman et al. found the genus *Actinomyces* increased with time, while *Actinomyces naeslundii* mainly increased after removal of the appliances^16^. Tanner et al. reported *Actinomyces* to be associated with gingivitis caused by orthodontic treatment^26^. These diverse results indicated that the genus *Actinomyces* showed variable behaviors by multiple factors such as host age on genus level. *Neisseria* has been reported to became higher in abundance twelve weeks after and became lower over time to the removal, and to be associated with low gingivitis group of orthodontic patients^16,26^. Most of these organisms that significantly decreased over time in plaque are aerobes or facultative anaerobes and are not associated with periodontal diseases. Commensal oral bacteria, such as *Neisseria* and *Haemophilus* spp., have previously been shown to decrease with time.

In our study, the genus *Streptococcus* also decreased over time both in plaque and saliva, although this difference was not statistically significant. Lucchese et al. reported in their systematic review study that the increase of *S. mutans* and *Lactobacillus* was influenced by the placement of orthodontic appliances^39^. We analyzed the relative abundance of bacteria in plaque and saliva by NGS, in contrast, their studies analyzed the whole volume of bacteria or bacterial DNA in plaque by colony counting and PCR. These methodological differences may account for the different conclusion.

**Figure 4:**
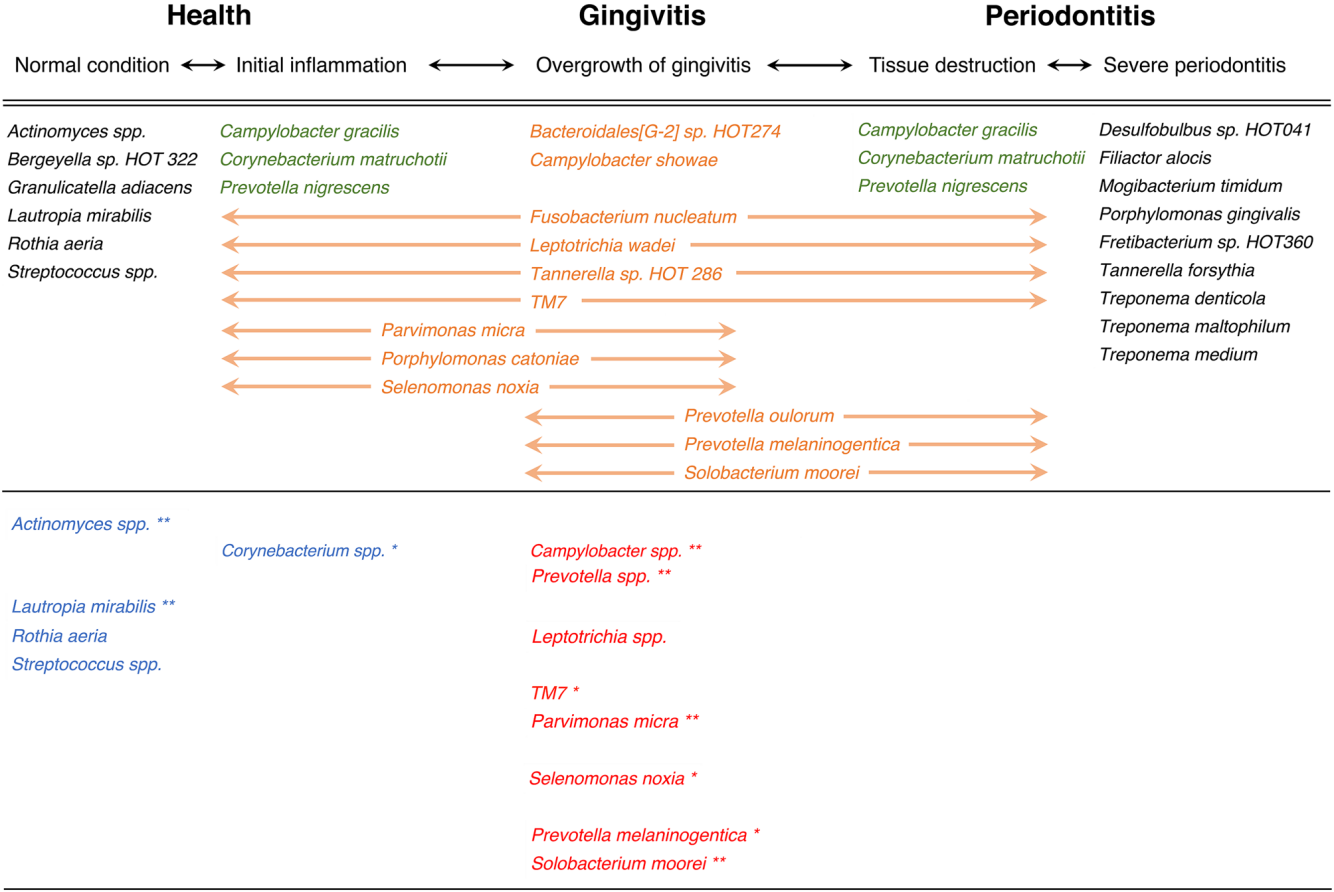
Comparison with previous findings on the gingival microbiome transition between health to periodontitis. The upper row shows the transitions of gingival condition. The middle row shows the model of temporal shifts in the gingival microbiome presented by Diaz et al. (2016). Gingivitis-associated species appear in orange text, core species (no change in relative abundance from health to periodontitis) appear in green text, and other bacteria appear in black text. The lower row shows the bacteria for which we observed changes in abundance in this study. The bacteria that increased from T0 to T1 appear in red text, and those that decreased appear in blue text. Significant differences are indicated as *p < 0.05 and **p < 0.01 by Mann–Whitney U test. *T0* time point prior to placement of fixed orthodontic appliance, *T1* time point approximately 6 months after the start of orthodontic treatment.

A recent study reported that the subgingival microbiome associated with gingival and periodontal inflammation is unique and distinct from the health-associated microbiome; proposing that the gingivitis-associated species are mediators of the transition in the health-to-periodontitis microbiome^40,41^. The species of *Solobacterium moorei, Parvimonas micra, Selenomonas noxia*, and *P. melaninogentica* which showed significant increase in supragingival plaque in our study (Fig. 4) are concordant with the gingivitis-associated bacteria of their report. The observed significant decrease in *Actinomyces* spp. and *Rothia dentocariosa* in supragingival plaque in this study is suggestive of a shift away from a healthy microbiome (Table 1b). These results suggest that orthodontic appliances are likely to change the gingival microbiome to a transitional stage between health and periodontitis.

The genera *Fusobacterium, Tennerella, Leptotrichia*, and *Paludibacter*, all obligate anaerobes, significantly increased over time in saliva, although not in plaque (Table 1c). *Fusobacterium* and *Tannerella* are regarded as major pathogens in periodontal diseases; specifically, *Fusobacterium nucleatum* and *Tannerella forsythia* possess prominent pathogenic potential. *F. nucleatum* forms aggregates with other suspected pathogens in periodontal disease and, thus, acts as a bridge between early and late colonizers on the tooth surface^42,43^. *T. forsythia* is frequently isolated with *P. gingivalis* from cases of active, chronic periodontitis and co-aggregates with *F. nucleatum*, suggesting a process for enhancing colonization in the biofilm. The previous systematic review study conducted by PCR and culture method reported *T. forsythia* showed significant increase three months after the beginning of orthodontic treatment but the data was only focused on subgingival plaque^29^. The results of the study and our study including saliva sample strongly suggested the increase of the genus *Tannerella* in oral cavity after placement of orthodontic appliances. Zhao et al. reported that there were significant decrease of *Prevotella* abundance and overall microbial community structure did not change in saliva in spite of using NGS method as our study^44^. It is speculated that their contrast result to our finding of increased periodontal pathogens were due to the different types of orthodontic appliances; whereas we targeted fixed orthodontic appliances, they targeted removal orthodontic appliances.

In our study, the genus *Granulicatella* was significantly decreased in saliva, but there was a report conducted by PCR that *Granulicatella elegans* showed significantly higher detection rate in plaque at white-spot lesions group of orthodontic patients^26^. The reverse result may be caused by the differences of methodology and sampling site.

It is consistent both in plaque and saliva in terms of the increase of periodontal pathogens and anaerobes. The change in microbiome in supragingival plaque is likely to reflect a structural change, induced by placement of fixed orthodontic appliances on the tooth surfaces. Similar results were also observed with saliva samples because saliva passes through orthodontic appliances, both on the labial side and the lingual side and appliances were found to have no effect on unstimulated salivary flow rate^45^.

These changes suggest that obligate anaerobes and periodontopathogenic bacteria replaced aerobic and facultative anaerobic bacteria in the oral microbiome, especially in plaque, following placement of fixed orthodontic appliances. Sallum et al. and Yáñez-Vico et al. reported that periodontal pathogens can be significantly reduced after fixed orthodontic appliance removal^46,47^. This dysbiosis is expected to be reversed after removal and therefore a longer-term microbiome study taking into consideration the potential factors affecting this reversal may provide further insight.

In this study, we found the same tendency of the dynamics of Japanese oral microbiome not only in plaque but also in saliva by orthodontic treatment compared with previous studies although to varying degrees. In addition, we found that orthodontic appliances changed the plaque microbiome to the transitional condition between health to periodontitis by comparing with previous reports^40,41^. We used high-throughput sequencing focused on the16S rRNA gene, providing only taxonomical findings. However, the microbiome is understood to be affected by interspecies metabolic interactions^2^. Whole genome sequencing and/or metabolome analysis may provide a better understanding of the response of the oral microbiome following placement of fixed orthodontic appliances. We observed dynamic changes in the oral microbiome, with an increase in anaerobic pathogens, even though we did not see significant clinical manifestations of gingival status. Some of these anaerobes are known to be periodontopathogenic, but orthodontists normally do not monitor precise gingival status. Thus, we recommend that orthodontists should better monitor the results of periodontal examination from the beginning of treatment, even when patients are young and have no gingival problems. Evaluation of more subjects, a control group that did not receive orthodontic treatment and clinical parameters may help to verify if fixed orthodontic appliances pose a periodontal risk.

## Conclusions

The oral microbiome measured in plaque and saliva changed during orthodontic treatment using fixed appliances. The shift represented an increase in anaerobes and periodontal pathogens and a decrease in commensal bacteria. Specifically, we propose that this shift, particularly in the supragingival microbiome, represents a transition between health and periodontitis.

## Methods

### Ethics statement

This study was approved by the Independent Ethics Committee of Hiroshima University Hospital, Hiroshima, Japan (No. 1645). Verbal informed consent was obtained from all the patients following provision of a full explanation of the study. This study included subjects under the age of 18, in which case informed consent was obtained from the parent or legal guardian. All samples were anonymized following collection.

### Subjects and the types of orthodontic appliances

All subjects participating in this study were Japanese and underwent orthodontic treatment from July 2016 to April 2018 at the Department of Orthodontics, Hiroshima University Hospital (n = 71; Supplementary Table 1). The type of malocclusion of the subjects was anterior crowding. The inclusion criteria were: (1) a healthy systemic condition; (2) no receipt of antibiotics or other medicines before sampling; (3) no severe gingivitis, with a periodontal probing depth of less than 4 mm, or alveolar bone loss visible on panoramic X-ray; (4) no fixed restorations or removable dentures; (5) receipt of tooth brushing instruction (TBI) by dental hygienists before bracket placement and maintenance of proper oral hygiene. Although the oral hygiene status at the time of initial visit differed among patients, we repeated oral hygiene instruction until plaque control record (PCR) values fell under 20%. The standard edgewise system 0.018 inch slot brackets were directly bonded to the labial tooth surfaces and an arch wire composed of an alloy of nickel and titanium or cobalt and chrome were fixed to bracket slots using ligature wires.

### Sample collection and storage

Three time points were evaluated: (T0) immediately before placement of fixed orthodontic appliances, (T1) 6 months after the beginning of orthodontic treatment, and (T2) immediately after removal of the appliances (40 months after placement on average, ranged from 20 to 62). Supragingival plaque samples were collected from upper and lower anterior teeth at T0 and T1. The area of tooth is above gingival margin at T0 and between gingival margin and cervical side of the brackets at T1. The sampling area was isolated with sterile cotton rolls and saliva removed by gentle air drying; supragingival plaque samples were then collected with a sterilized explorer. Unstimulated saliva samples were collected into a clean paper cup at T0, T1, and T2. To ensure standardization, the subjects were instructed to avoid eating, drinking, and tooth brushing at least 2 h before the sample taking. All the samples were collected at the beginning of the visits and pooled approximately into a 1.5 mL microcentrifuge tube containing 0.5 mL of phosphate buffered saline (PBS) and stored immediately at − 80 °C until use^48^.

### DNA extraction

Microbial DNA was extracted from supragingival plaque and saliva samples using a MasterPure Complete DNA and RNA Purification Kit (Epicentre, Madison, WI, USA) according to the manufacturer’s instructions as previously performed by^49^, with minor modifications.

### Library preparation and 16S rRNA gene sequencing

The V1-V2 hypervariable regions of 16S rRNA gene sequences were amplified by PCR using KAPA HiFi HotStart ReadyMix (KAPA Biosystems, Wilmington, MA, USA) and primers from the Nextera XT Index Kit (Illumina, San Diego, CA, USA)^50^. Amplification was performed on a Veriti Thermal Cycler (Thermo Fisher Scientific, Waltham, MA, USA). PCR products (approximately 450 bp) were visualized by agarose gel electrophoresis, purified using Agencourt AMPure XP magnetic beads (Beckman Coulter, Brea, CA, USA), and DNA concentrations were measured using a NanoDrop ND1000 spectrophotometer and Invitrogen Qubit 4.0 Fluorometer (Thermo Fisher Scientific). Tag-indexed samples were diluted and pooled into a low DNA-binding tube in equal amounts from each sample. DNA concentration in the pooled samples was confirmed by qPCR, using KAPA SYBR FAST qPCR MasterMix (KAPA Biosystems). The library was applied to a MiSeq sequencing platform (Illumina). Nucleotide sequence data reported are available in the DNA Data Bank of Japan (DDBJ) Sequenced Read Archive under the accession number DRA010713.

### Sequence data analysis and statistical analysis

Sequence data were analyzed with CLC Genomics Workbench ver.9 (QIAGEN, Venlo, Netherlands). Sequences were assigned to the same operative taxonomic units (OTUs) when within 97% similarity, in reference to the Greengenes database (https://greengenes.secondgenome.com). Additional statistical analysis such as Mann–Whitney U test and Kruskal–Wallis test were conducted using StatView 5.0 J (SAS Institute, Cary, NC, USA). Mann–Whitney U test was used for comparisons between two groups and Kruskal–Wallis test was used in multiple comparisons.

### Ethic approval

All procedures performed in this study involving human participants were in accordance with the ethical standards of the institutional research committee and with the 1964 Helsinki Declaration and its later amendment or comparable ethical standards.

## Supplementary Information

Supplementary Information
