# Supplementary material for: The impact of fixed orthodontic appliances on oral microbiome dynamics in Japanese patients
Source: Sci Rep. 2020 Dec 15;10:21989. doi: 10.1038/s41598-020-78971-2 (PMC7738506; doi:10.1038/s41598-020-78971-2)
Supplement: Supplementary file 1 — Supplementary Information [file 41598_2020_78971_MOESM1_ESM.pdf]

[The title of the manuscript]

The impact of fixed orthodontic appliances on oral microbiome dynamics in Japanese patients

[Full authors list]

Isamu Kado[1,2], Junzo Hisatsune[2,4], Keiko Tsuruda[2,3], Kotaro Tanimoto[1] , Motoyuki Sugai[2,4*]

[1] Department of Orthodontics and Craniofacial Developmental Biology, Hiroshima University Graduate School of Biomedical & Health Sciences, Hiroshima, Japan

[2] Department of Antimicrobial Resistance, Hiroshima University Graduate School of Biomedical & Health Sciences, Hiroshima, Japan

[3]Department of Oral Epidemiology, Hiroshima University Graduate School of Biomedical & Health Sciences, Hiroshima, Japan

[4] Antimicrobial Resistance Research Center, National Institute of Infectious Diseases, Tokyo,

Japan

Supplementary Table 1. Patients' characteristics. Number of patients, sex and age

	T0 Group			T1 Group			T2 Group		
	Female	Male	Total	Female	Male	Total	Female	Male	Total
Number of patients, n	22	6	28	19	7	26	14	3	17
Age, y (mean ± SD)	22.6 ± 8.4	20.2 ± 7.9	22.1 ± 8.2	22.1 ± 8.0	19.8 ± 7.4	21.3 ± 7.8	31.6 ± 12.9	18.8 ± 2.7	29.3 ± 12.3

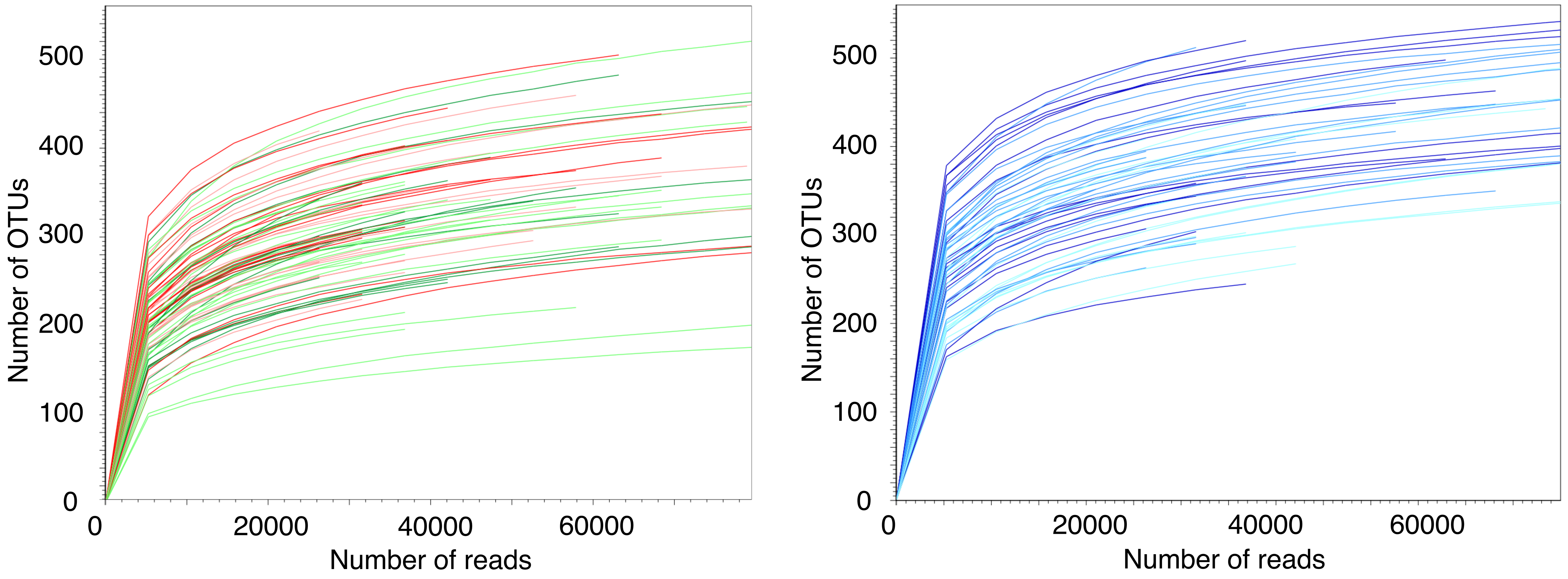

Supplementary Figure 1. Alpha diversity of the oral microbiome following placement of fixed orthodontic devices
Alpha diversity of supragingival plaque (a) and saliva (b). In supragingival plaque (a), green and red lines represent T0 and T1 samples, respectively, where dark lines represent upper teeth and lighter lines represent lower teeth. In saliva (b), dark, middle and light blue lines represent T0, T1, and T2 samples respectively. T0, time point prior to placement of fixed orthodontic appliance; T1, time point approximately 6 months after the start of orthodontic treatment; T2, time point after appliance removal (average, 40 months after placement). These images were generated by CLC Genomics Workbench ver.9 (QIAGEN, Venlo, Netherlands) (https://www.qiagen.com/jp/products/discovery-and-translational-research/next-generation-sequencing/informatics-and-data/analysis-and-visualization/clc-genomics-workbench/#orderinginformation).
